# Cognitive Impairment and Functional Independence in Hospitalized Patients with Atrial Fibrillation: A Cross-Sectional Study

**DOI:** 10.3390/jcm15208075

**Published:** 2026-10-17

**Authors:** Dania Hasanein, Oana Elena Țunea, Samuel Ardelean, Daniel Florin Lighezan

**Affiliations:** 1 Doctoral School of Medicine, “Victor Babeș” University of Medicine and Pharmacy, Eftimie Murgu Square No. 2, 300041 Timișoara, Romania; ancusa.oana@umft.ro (O.E.Ț.); samuel.ardelean@umft.ro (S.A.); dlighezan@umft.ro (D.F.L.); 2 Centre for Advanced Research in Cardiovascular Pathology and Hemostaseology, “Victor Babeș” University of Medicine and Pharmacy, Eftimie Murgu Square No. 2, 300041 Timișoara, Romania; 3 Department of Internal Medicine I, 1st Clinic of Medical Semiology, “Victor Babeș” University of Medicine and Pharmacy, Eftimie Murgu Square No. 2, 300041 Timișoara, Romania; 4 Department of Cardiology, Institute of Cardiovascular Diseases, Str. Gheorghe Adam No. 13A, 300310 Timișoara, Romania; 5 Department V, Internal Medicine 1, “Victor Babeș” University of Medicine and Pharmacy, Eftimie Murgu Square No. 2, 300041 Timișoara, Romania; 6 Multidisciplinary Heart Research Center, Faculty of Medicine, “Victor Babeș” University of Medicine and Pharmacy, Eftimie Murgu Square No. 2, 300041 Timișoara, Romania

**Keywords:** atrial fibrillation, cognitive impairment, ischemic stroke, CHA_2_DS_2_-VASc score, Montreal Cognitive Assessment, preventive cardiology

## Abstract

**Background/Objectives:** Atrial fibrillation (AF) is associated with cognitive decline even without clinically apparent stroke, but the relative contributions of demographic, vascular, inflammatory and therapeutic factors remain unclear. We aimed to explore clinical determinants of cognitive and functional performance in hospitalized patients with AF. **Methods:** In this exploratory cross-sectional study, 62 consecutive patients with documented AF admitted to an internal medicine department underwent assessment with the Montreal Cognitive Assessment (MoCA), Mini-Mental State Examination (MMSE) and Activities of Daily Living (ADL) scale. Associations were examined with multivariable linear regression, with false-discovery-rate correction and sensitivity analyses. **Results:** Mean age was 75.3 ± 8.1 years; 17 patients (27.4%) had a previous ischemic stroke. Fifty-nine patients (95.2%) scored below the MoCA threshold of 26 and 35 (56.5%) below the MMSE threshold of 24. Patients with previous stroke scored lower on all three instruments (MoCA mean difference 4.13, 95% CI 0.75–7.51; MMSE 4.84, 1.67–8.01; ADL 1.69, 0.25–3.12). After correction for multiple testing, age remained associated with all three outcomes and previous stroke with MMSE score; no association was detected for sex, education, diabetes, C-reactive protein, LDL-cholesterol or anticoagulant type. Higher CHA_2_DS_2_-VAScscores were associated with poorer performance, though not independently of their own components. **Conclusions:** In this exploratory study, cognitive impairment was common among hospitalized patients with AF and was associated with age and previous stroke. These hypothesis-generating findings require confirmation in larger prospective studies.

## 1. Introduction

Atrial fibrillation (AF) is the most common sustained cardiac arrhythmia worldwide and carries a broad burden of cardiovascular and thromboembolic complications, including ischemic stroke, heart failure, silent cerebral infarction and systemic thromboembolism, which together contribute to the excess morbidity and mortality seen in this population [1]. Cognitive impairment is increasingly recognized as a further complication: patients with AF have a higher risk of mild cognitive impairment and dementia than those without, even in the absence of clinically apparent stroke [2].

Several determinants of cognitive impairment in AF have been proposed. AF and cognitive decline share risk factors, among them older age, hypertension, diabetes mellitus and heart failure, and the proposed mechanisms include silent cerebral infarction, chronic cerebral hypoperfusion, endothelial dysfunction and cerebral small-vessel disease. Systemic inflammation, reflected by elevated C-reactive protein (CRP), has been associated with atrial remodeling and an increased risk of thromboembolic events [3]. Whether oral anticoagulation influences cognition beyond stroke prevention remains uncertain, although current guidelines recommend direct oral anticoagulants (DOACs) as first-line therapy for most patients with non-valvular AF [1,4]. Higher CHA_2_DS_2_-VASc scores, used routinely to estimate thromboembolic risk, have also been associated with incident dementia [5] and with a greater burden of white matter hyperintensities and physical frailty [6].

Identifying which of these factors are associated with cognitive and functional impairment matters for preventive cardiology, because cognitive dysfunction undermines treatment adherence, functional independence and long-term prognosis. Much of the existing evidence comes from studies of dementia incidence; fewer studies have examined hospitalized patients, in whom cognitive screening could be undertaken during routine care, or have assessed cognitive and functional performance together while accounting for educational attainment, a major determinant of performance on screening instruments. The present study was conducted in the population served by our department in Timișoara, Romania.

This exploratory cross-sectional study of a consecutive sample of hospitalized patients with AF therefore aimed to describe the prevalence of cognitive impairment and to explore its clinical correlates. No hypotheses were prespecified. Candidate correlates were selected from the literature and grouped as demographic factors (age, sex and educational attainment), cerebrovascular and metabolic factors (previous ischemic stroke and diabetes mellitus), biomarkers of the proposed inflammatory and lipid pathways (CRP and low-density lipoprotein cholesterol) and anticoagulant therapy. Cognitive function was assessed with the Montreal Cognitive Assessment (MoCA) and the Mini-Mental State Examination (MMSE), and functional status with the Activities of Daily Living (ADL) scale.

## 2. Materials and Methods

### 2.1. Study Design and Population

This cross-sectional observational study was conducted in the Department of Internal Medicine, Timișoara Municipal Emergency Clinical Hospital, between January and May 2026. Consecutive patients admitted with atrial fibrillation were screened. Eligible participants were adults with electrocardiographically documented AF who were receiving oral anticoagulant therapy and were able to complete cognitive and functional assessments. Patients who declined to participate, who were not receiving an oral anticoagulant, whose condition prevented reliable cognitive evaluation, or whose clinical data were incomplete were excluded.

The study was conducted in accordance with the Declaration of Helsinki and approved by the Institutional Ethics Committee of the “Victor Babeș” University of Medicine and Pharmacy, Timișoara (Approval No. 93/02.10.2023 rev.2025). All participants gave written informed consent.

### 2.2. Clinical Assessment

Demographic and clinical data were collected from medical records and patient interviews. Recorded variables were age, sex, AF subtype (first diagnosed, paroxysmal, persistent, permanent or pharmacologically converted), previous ischemic stroke, diabetes mellitus, anticoagulant treatment, statin therapy, serum C-reactive protein (CRP) and low-density lipoprotein cholesterol (LDL-C). Pharmacologically converted AF denoted AF restored to sinus rhythm with antiarrhythmic medication before assessment. The CHA_2_DS_2_-VASc score was calculated for every patient from its standard components. Educational attainment was recorded in four categories (middle school, professional school, high school, university) and converted, for correlation and regression analyses, to the corresponding number of years of formal schooling under the Romanian education system (8, 10, 12 and 16 years, respectively). Place of residence was classified as rural or urban. Left ventricular ejection fraction (LVEF) was taken from the transthoracic echocardiogram performed during admission; because a minority of reports specified a range rather than a single value, LVEF is presented categorically (<40%, 40–49%, ≥50%).

### 2.3. Cognitive Assessment

Cognitive function was evaluated with the Montreal Cognitive Assessment (MoCA) and the Mini-Mental State Examination (MMSE), both administered by trained physicians during hospitalization under standardized conditions. Each is a validated 30-point screening instrument covering multiple cognitive domains, with higher scores indicating better performance.

### 2.4. Functional Assessment

Functional status was assessed using the Activities of Daily Living (ADL) scale in routine institutional use at our center. The instrument rates five basic self-care activities—personal hygiene, dressing, toileting, sphincter continence and feeding—each on a three-point ordinal scale (2 = independent, 1 = requires partial assistance, 0 = dependent). Item scores are summed to give a total ranging from 0 to 10, with higher scores indicating greater functional independence. The total score was analyzed as a continuous variable.

### 2.5. Laboratory Assessment

CRP and LDL-C were measured in fasting blood samples taken as part of the routine clinical evaluation during hospitalization, and analyzed in the hospital’s central laboratory using standardized methods.

### 2.6. Statistical Analysis

Analyses were performed in IBM SPSS Statistics version 30 (IBM Corp., Armonk, NY, USA). Distributional assumptions were checked with the Shapiro–Wilk test and inspection of histograms and Q–Q plots. Continuous variables are presented as mean ± standard deviation (SD) or median with interquartile range (IQR) as appropriate, and categorical variables as frequencies and percentages. Continuous variables were compared with the independent-samples *t*-test or the Mann–Whitney U test according to distribution, and categorical variables were compared with the chi-square test. All tests were two-sided and *p* < 0.05 was considered significant.

For the comparison according to previous ischemic stroke, mean differences are reported with 95% confidence intervals and effect sizes as Cohen’s d based on the pooled SD. Associations between biomarkers and outcomes were assessed with Spearman’s rank correlation. Scores according to the prescribed DOAC agent are presented descriptively; because of the small edoxaban and rivaroxaban groups, no statistical comparison between agents was made.

The association between the CHA_2_DS_2_-VASc score and each outcome was assessed by Spearman’s rank correlation and by separate linear regression models. Because age, diabetes mellitus, previous stroke and female sex are themselves components of the score, they were not entered as covariates alongside it; the score was modeled separately, unadjusted and adjusted for the two available non-component variables (CRP and LDL-C). To explore independent predictors of cognitive and functional performance, separate multiple linear regression models were fitted with MoCA, MMSE and ADL scores as dependent variables, and age, sex, educational attainment, previous ischemic stroke, diabetes mellitus, CRP, LDL-C and anticoagulant therapy as independent variables. These variables were chosen on the basis of the prior literature, as described in the Introduction Section, rather than by statistical selection. Regression coefficients (β) are reported with 95% confidence intervals, and model performance as R^2^, adjusted R^2^ and root mean square error (RMSE).

To account for multiple testing, *p*-values were adjusted with the Benjamini–Hochberg false-discovery-rate procedure, treating the tests reported in each table as a family, and adjusted values (q-values) are reported alongside unadjusted *p*-values. Regression assumptions were assessed using variance inflation factors, the Breusch–Pagan test for heteroscedasticity, the Shapiro–Wilk test of residual normality, the RESET test for non-linearity and Cook’s distance for influential observations; heteroscedasticity-robust (HC3) standard errors and bootstrap percentile confidence intervals were additionally estimated.

Because the ratio of predictors to participants was limited, the stability of the multivariable models was examined in sensitivity analyses: a parsimonious model including age, previous ischemic stroke, educational attainment and sex; LASSO and ridge regression on standardized predictors, with the LASSO penalty selected by repeated five-fold cross-validation; bootstrap resampling (2000 resamples) of the coefficient estimates; and bootstrap optimism correction of R^2^. Further models added AF subtype (permanent versus non-permanent) and LVEF below 40%. CRP was additionally analyzed after log transformation and after exclusion of participants with CRP above 10 mg/L, as higher values may reflect an acute-phase response, and the association of place of residence with each outcome was examined with adjustment for age, educational attainment and previous ischemic stroke.

The study was exploratory, and no hypotheses were prespecified. The study size was determined by the number of eligible patients admitted during the recruitment period rather than by an a priori power calculation, so the final sample of 62 represents a consecutive convenience sample. Patients were enrolled consecutively rather than selectively, assessments were performed by the same trained examiner using standardized instructions, and educational attainment was recorded and entered into the multivariable models. Because participants were recruited during hospital admission, selection bias toward greater impairment than in a community-dwelling AF population cannot be excluded, and assessment during an acute admission may underestimate baseline performance. No data were missing for any analyzed variable, so no imputation was required.

## 3. Results

### 3.1. Study Population

**Figure 1:**
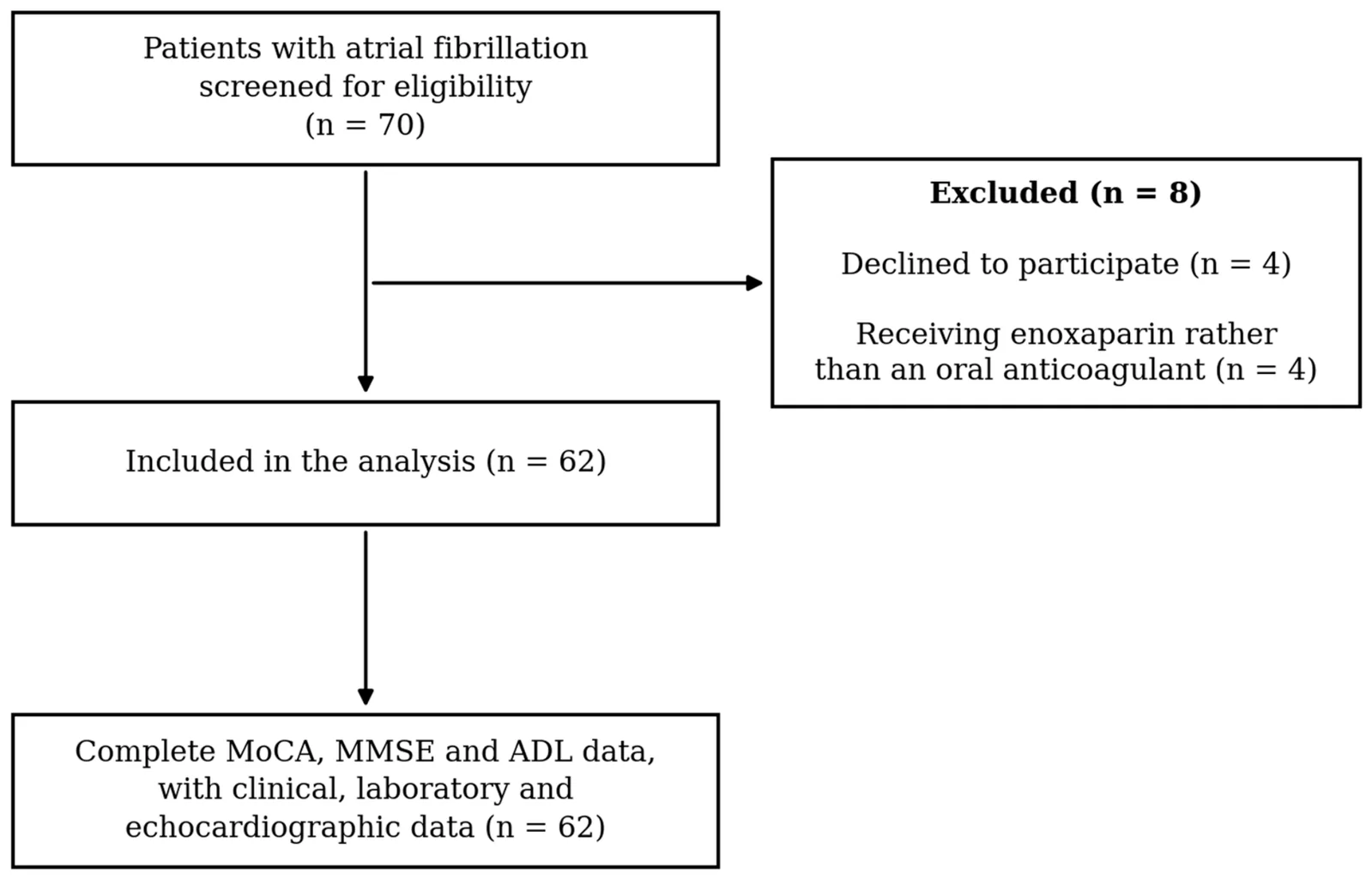
Flow of participants through the study. Of 70 patients with atrial fibrillation screened for eligibility, 8 were excluded (4 declined to participate and 4 were receiving enoxaparin rather than an oral anticoagulant), leaving 62 patients with complete cognitive, functional, clinical, laboratory and echocardiographic data.

Seventy patients with atrial fibrillation were screened for eligibility. Eight were excluded—four declined to participate and four were receiving enoxaparin rather than an oral anticoagulant—leaving 62 participants (Figure 1). Thirty-four (54.8%) were women, and the mean age was 75.32 ± 8.08 years, women being significantly older than men (77.82 ± 6.98 vs. 72.29 ± 8.52 years, *p* < 0.01). Permanent atrial fibrillation was the most frequent subtype (54.8%), and the distribution of subtypes did not differ by sex (*p* = 0.23). Previous ischemic stroke was documented in 17 patients (27.4%) and was more common in women than in men (41.2% vs. 10.7%, *p* < 0.01); diabetes mellitus was present in 18 (29.0%). Serum CRP was higher in men than in women (median 12.6, IQR 4.8–62.1 vs. 4.7, IQR 1.8–11.6 mg/L, *p* = 0.02), whereas LDL-C did not differ by sex. Approximately 90% of patients were receiving statin therapy. Most patients (91.9%) received a direct oral anticoagulant. Baseline characteristics are summarized in Table 1.

Cognitive screening scores were low across the cohort. Fifty-nine patients (95.2%, 95% CI 86.5–99.0) scored below the conventional MoCA threshold of 26 points and 30 (48.4%) below 18, indicating moderate-to-severe impairment. On the MMSE, 35 patients (56.5%, 95% CI 43.3–69.0) scored below 24 points and 16 (25.8%) below 18. MMSE impairment was more frequent among patients with previous ischemic stroke (82.4% vs. 46.7%, *p* = 0.011). The difference between the proportions identified by the two instruments is consistent with the lower sensitivity of the MMSE to vascular cognitive impairment.

### 3.2. Previous Stroke and Cognitive and Functional Performance

Compared with patients without previous stroke, those with a history of stroke scored lower on the MoCA (mean difference 4.13, 95% CI 0.75 to 7.51; *p* = 0.02), the MMSE (4.84, 95% CI 1.67 to 8.01; *p* < 0.01) and the ADL scale (1.69, 95% CI 0.25 to 3.12; *p* = 0.02), with moderate-to-large effect sizes (Cohen’s d 0.70, 0.87 and 0.67, respectively). The confidence intervals were wide and extended close to the null, reflecting the limited size of the previous-stroke subgroup (*n* = 17) (Table 2). All three differences remained significant after false-discovery-rate correction (q ≤ 0.022).

### 3.3. Inflammatory and Lipid Biomarkers

Serum CRP correlated weakly and negatively with ADL score (ρ = −0.31, *p* = 0.015, q = 0.039), although this correlation was borderline (q = 0.056) when the three correlations between the outcome instruments were excluded from the family. No association was detected between CRP and MoCA or MMSE scores, or between LDL-C and any outcome. In sensitivity analyses, log-transformed CRP was independently associated with ADL score (β per log unit = −0.43, 95% CI −0.82 to −0.05, *p* = 0.027) and showed a borderline association with MMSE score (*p* = 0.067); after exclusion of participants with CRP above 10 mg/L (*n* = 37), the correlation between CRP and ADL score was stronger (ρ = −0.43, *p* = 0.008), and no association was detected with MoCA or MMSE scores. The two cognitive instruments were strongly correlated with each other (ρ = 0.86, *p* < 0.001), and both correlated moderately with ADL score (ρ = 0.49 and 0.50, both *p* < 0.001), indicating that poorer cognitive performance accompanied reduced functional independence. The complete correlation analysis is presented in Table 3.

### 3.4. Oral Anticoagulant Therapy

**Figure 2:**
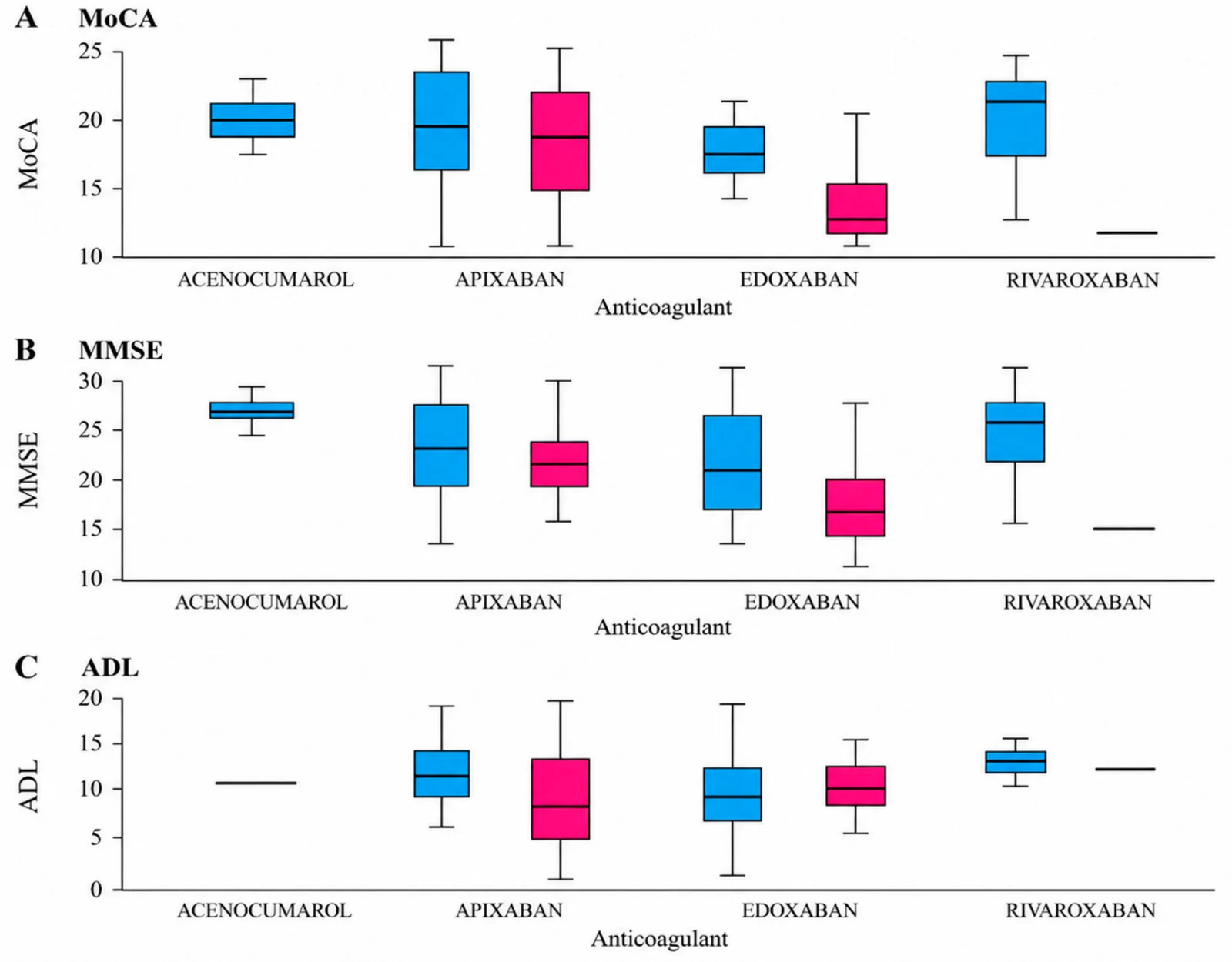
Cognitive and functional performance according to oral anticoagulant therapy and previous ischemic stroke status. (**A**) Montreal Cognitive Assessment (MoCA), (**B**) Mini-Mental State Examination (MMSE), and (**C**) Activities of Daily Living (ADL) scores according to oral anticoagulant therapy (acenocoumarol, apixaban, edoxaban, and rivaroxaban), stratified by the presence (1) or absence (0) of previous ischemic stroke. Boxplots display the median (horizontal line), interquartile range (box), and minimum and maximum values (whiskers). Individual data points outside the whiskers represent outliers. AVC denotes previous ischemic stroke (0 = absent; 1 = present). **Note:** For rivaroxaban, patients with previous ischemic stroke (1) are represented by a single observation (horizontal line) because of the small sample size.

Scores according to the prescribed anticoagulant are presented descriptively. The groups were of markedly unequal size (apixaban *n* = 41, edoxaban *n* = 12, rivaroxaban *n* = 4, acenocoumarol *n* = 5), and with 57 patients across the three DOAC groups the study had approximately 36% power to detect a medium effect (Cohen’s f = 0.25); no statistical comparison was therefore made. Mean MoCA scores were 18.05 ± 6.13 with apixaban, 14.17 ± 5.84 with edoxaban and 16.25 ± 9.00 with rivaroxaban; mean MMSE scores were 21.71 ± 5.90, 18.42 ± 5.70 and 20.25 ± 7.50; and mean ADL scores were 7.93 ± 2.66, 6.58 ± 2.91 and 9.75 ± 0.50, respectively (Figure 2). Because the comparison was underpowered, no conclusion about equivalence or difference between agents can be drawn.

### 3.5. Multivariable Predictors

Separate multiple linear regression models were fitted for MoCA, MMSE and ADL scores, with age, sex, educational attainment, previous ischemic stroke, diabetes mellitus, CRP, LDL-C and oral anticoagulant therapy as independent variables (Table 4 and Table 5). Age was associated with lower scores on all three instruments, and these associations remained after false-discovery-rate correction (q ≤ 0.023). Previous ischemic stroke was associated with lower MMSE score (q = 0.030); its associations with MoCA score (*p* = 0.027, q = 0.130) and ADL score (β = −1.36, 95% CI −2.93 to 0.20, *p* = 0.09) were consistent in direction but did not survive correction. No association was detected for sex, education, diabetes mellitus, CRP, LDL-C or anticoagulant therapy.

In sensitivity analyses (Supplementary Tables S1–S4 and S6), age was retained in every model and in 97–100% of bootstrap LASSO fits, and its coefficient was negative in all bootstrap resamples. The coefficient for previous stroke was negative in at least 92% of resamples but statistically significant in only 38–79%, indicating a consistent direction but limited precision. Bootstrap optimism correction reduced R^2^ from 0.35 to 0.16 for MoCA, from 0.42 to 0.25 for MMSE and from 0.27 to 0.06 for ADL (Table 5), indicating that the models describe individual associations rather than providing useful prediction. Adding permanent AF or LVEF below 40% did not attenuate the associations of age or previous stroke, and LVEF below 40% was associated with lower ADL score (β = −1.83, 95% CI −3.63 to −0.02). Diagnostics showed no heteroscedasticity or influential observations; residuals were non-normal for ADL, reflecting a ceiling effect, and robust and bootstrap estimates led to the same conclusions.

### 3.6. CHA_2_DS_2_-VASc Score

The median CHA_2_DS_2_-VASc score was 5 (interquartile range 5–7; range 1–9), and higher scores were associated with poorer performance on all three instruments (Table 3; q ≤ 0.017). In unadjusted linear regression, each one-point increase corresponded to a 1.30-point-lower MoCA score (95% CI −2.16 to −0.44), a 1.29-point-lower MMSE score (95% CI −2.11 to −0.46) and a 0.57-point-lower ADL score (95% CI −0.93 to −0.20), all *p* ≤ 0.004 and q ≤ 0.004; these associations were essentially unchanged after adjustment for CRP and LDL-C (Table 6).

Because age, diabetes mellitus, previous ischemic stroke and female sex are constituent components of the score, it was not entered into the models reported in Table 4. When forced into a model alongside age and previous stroke, the association was abolished (MoCA β = +0.60, *p* = 0.41; MMSE β = +0.96, *p* = 0.15; ADL β = −0.03, *p* = 0.94), while age and previous stroke retained their associations. Variance inflation factors remained below 3.4, indicating that the loss of significance reflects the score being a coarser summary of the same underlying information rather than unstable estimation.

### 3.7. Education, Residence and Left Ventricular Function

Educational attainment was low overall: 36 patients (58.1%) had completed professional school and seven (11.3%) middle school only, whereas 17 (27.4%) had completed high school and two (3.2%) held a university degree. Years of schooling correlated with MMSE score (ρ = 0.30, *p* = 0.019, q = 0.042) and, at borderline significance, with MoCA score (ρ = 0.24, *p* = 0.057), but no association was detected with ADL score. Education was inversely related to age (ρ = −0.33, *p* = 0.008) and no independent association with any outcome was detected in the multivariable models (Table 4).

Thirty-nine patients (62.9%) resided in rural areas. Rural residents had fewer years of schooling than urban residents (10.10 ± 1.29 vs. 11.22 ± 1.78 years, *p* = 0.017) and lower MoCA scores (16.21 ± 5.83 vs. 19.13 ± 6.41, *p* = 0.045), with no difference in MMSE or ADL scores, age or CHA_2_DS_2_-VASc score. After adjustment for age, educational attainment and previous ischemic stroke, rural residence remained associated with lower MoCA score (β = −4.15, 95% CI −7.13 to −1.18, *p* = 0.007), whereas no association was detected with MMSE or ADL scores (Supplementary Table S7). Left ventricular ejection fraction was below 40% in 11 patients (17.7%), 40–49% in 25 (40.3%) and 50% or above in 26 (41.9%), was lower in men than in women (*p* < 0.01), and correlated weakly with ADL score only (ρ = 0.26, *p* = 0.039), a correlation that did not survive false-discovery-rate correction (q = 0.077).

## 4. Discussion

In this exploratory cross-sectional study of hospitalized patients with atrial fibrillation, previous ischemic stroke and advancing age were the only independent predictors of cognitive performance. This is consistent with cohort studies identifying both as among the strongest determinants of incident cognitive impairment and dementia in AF: Kalantarian et al. reported that AF increases dementia risk even after adjustment for traditional vascular risk factors, and Friberg and Rosenqvist identified previous stroke as an important determinant of subsequent cognitive decline [7,8]. Aging promotes atrial remodeling, endothelial dysfunction, vascular stiffness and cerebral small-vessel disease while reducing neuronal reserve, so older patients with AF may be particularly susceptible to cumulative cerebral injury [9], although our data cannot address these mechanisms.

In unadjusted comparisons, patients with previous stroke scored lower on both cognitive instruments and on the ADL scale. If confirmed, this would matter clinically, because cognitive impairment in AF may be accompanied by deterioration in everyday functioning that affects medication adherence, self-management and long-term cardiovascular outcomes. Systematic cognitive assessment after ischemic stroke could therefore help identify patients requiring additional multidisciplinary support, a possibility that requires prospective evaluation.

No association was detected between LDL-C and any outcome, or between CRP and cognitive scores; given the sample size, these findings cannot exclude associations of modest magnitude. CRP was, however, associated with functional status, and this association was stronger after exclusion of probable acute-phase values, consistent with the possibility that acute inflammation obscures a relationship between lower-grade inflammation and function. Previous observational studies have reported weak or non-significant associations between single CRP measurements and cognition after adjustment for vascular risk factors [10], and a single in-hospital measurement cannot distinguish acute from chronic inflammatory activity. For LDL-C, previous findings are conflicting [11,12], and because approximately 90% of our patients were receiving statins, a single in-hospital measurement is likely to reflect treatment rather than long-term exposure.

No independent association between anticoagulant therapy and cognitive performance was detected, and scores according to individual DOAC agents were presented descriptively only. With small edoxaban, rivaroxaban and vitamin K antagonist groups, the study was not powered to compare anticoagulant strategies, and no conclusion about their equivalence or difference can be drawn. Registry data suggest that DOAC therapy overall may be associated with a lower incidence of dementia than vitamin K antagonists [13,14], a question that requires adequately powered prospective studies.

Cognitive performance correlated closely with functional independence: patients with lower MoCA and MMSE scores consistently had lower ADL scores. Notably, the two instruments identified very different proportions as impaired (95.2% by MoCA versus 56.5% by MMSE), consistent with reports that the MoCA is more sensitive than the MMSE to the subtle deficits characteristic of vascular cognitive impairment [15], and supporting its use for routine screening in this population.

The CHA_2_DS_2_-VASc score, calculated routinely for every patient with AF to guide anticoagulation, was inversely associated with both cognitive and functional performance, consistent with work linking higher scores to white matter hyperintensity burden and physical frailty [6]. Practically, a score already available at the bedside may help identify patients who warrant cognitive screening without additional investigation. The association is not independent, however: age, diabetes mellitus, previous stroke and female sex are components of the score, and in our data it carried no predictive information beyond the age and cerebrovascular history from which it is derived.

Educational attainment is among the strongest determinants of performance on brief cognitive tests, and its omission is a recognized weakness of much of the literature in this field. In our cohort, education correlated with MMSE score on univariable analysis but no independent association was detected once age was taken into account, the two being inversely related. Place of residence showed a different pattern: rural patients scored lower on the MoCA, and this difference persisted after adjustment for age, years of schooling and previous stroke. Years of schooling therefore did not explain it, and the association may reflect factors that were not measured, such as the quality of education, socioeconomic circumstances or access to healthcare. As a post hoc finding, it should be regarded as hypothesis-generating, but it argues for cautious interpretation of absolute screening scores in this population and for applying education-adjusted cut-offs where they exist.

These findings should be read as exploratory. Multiple comparisons were made across three outcomes and a range of clinical, biochemical and echocardiographic variables. False-discovery-rate correction was applied within each table, but results close to the significance threshold depended on how the family of tests was defined, and the associations reported for CHA_2_DS_2_-VASc score, education, residence, LVEF and anticoagulant agent are hypothesis-generating and require replication.

Taken together, our findings are consistent with the concept that prevention of cognitive decline in AF may need to extend beyond anticoagulation alone, to include control of vascular risk factors and early identification of cognitive impairment. Whether such an approach improves outcomes cannot be determined from a cross-sectional study and requires prospective evaluation.

### 4.1. Strengths and Limitations

This study has several strengths. Cognitive performance was evaluated using two validated screening instruments (MoCA and MMSE), while functional status was simultaneously assessed using the ADL scale, providing a broader evaluation of patient outcomes. Furthermore, multiple clinical variables relevant to cognitive decline, including previous ischemic stroke, inflammatory status, lipid profile, diabetes mellitus, age, sex, educational attainment, place of residence, left ventricular ejection fraction, CHA_2_DS_2_-VASc score and anticoagulant therapy, were analyzed within the same cohort.

Several limitations should also be acknowledged. First, the cross-sectional design precludes conclusions regarding causality or temporal relationships between atrial fibrillation and cognitive decline. Second, the study was conducted at a single center with a relatively modest sample size; no a priori power calculation was performed and no hypotheses were prespecified, which limits statistical power and generalizability. Third, cognitive performance was assessed using screening instruments rather than comprehensive neuropsychological testing, and functional status was measured with a five-item institutional ADL scale rather than an internationally validated instrument such as the Katz Index or the Barthel Index, which limits direct comparison of absolute ADL scores with those reported elsewhere. Educational attainment was recorded as the highest level completed rather than as exact years of schooling, and its distribution in this cohort was narrow, with only two participants having completed tertiary education; this limits the precision with which its contribution could be estimated. Residual confounding cannot be excluded because other potentially important variables, including duration of atrial fibrillation, heart-rate control at admission, a clinical diagnosis of heart failure, medication adherence and neuroimaging markers of cerebral small-vessel disease, were not systematically available. Frailty was not assessed with a validated instrument; although the ADL scale captures dependence in basic self-care, it is not a substitute for a formal frailty assessment. CRP and LDL-C were each measured once during admission, so CRP may reflect acute-phase responses, and LDL-C, measured in a population largely receiving statins, may poorly reflect long-term exposure. The ADL scale showed a ceiling effect, with 22 of 62 participants achieving the maximum score. The small number of patients receiving rivaroxaban, edoxaban and vitamin K antagonists left the comparison between anticoagulant strategies substantially underpowered, and the null findings for anticoagulant agent should therefore not be read as evidence of equivalence. The ratio of predictors to observations in the multivariable models (eight predictors for 62 participants) similarly limits the precision of the estimates. Although simulation studies suggest that linear regression requires only about two subjects per variable for adequate estimation of coefficients [16], bootstrap optimism correction showed that the explained variance of our models was substantially overestimated, and these models are best regarded as exploratory. Multiple testing increases the risk of false-positive findings; false-discovery-rate correction was applied, but results near the threshold were sensitive to the definition of the test family. Finally, the direction of the principal potential biases can be anticipated. Recruitment during hospital admission is likely to have inflated the absolute prevalence of cognitive impairment relative to a community-based AF population, while leaving the internal comparisons between patients with and without previous stroke largely unaffected. Residual confounding by unmeasured cerebrovascular burden would tend to exaggerate the apparent effect of previous stroke, whereas the coarse categorization of education would tend to underestimate its contribution; the observed association between previous stroke and cognitive performance may therefore be smaller than the point estimates suggest.

### 4.2. Clinical Implications

If confirmed, the present findings would support incorporating cognitive evaluation into the routine assessment of patients with atrial fibrillation, particularly among older individuals and those with a history of ischemic stroke. Early recognition of cognitive impairment may improve treatment adherence, facilitate shared decision-making and enable timely implementation of multidisciplinary interventions aimed at preserving functional independence. Future prospective multicenter studies with larger patient populations are warranted to clarify the long-term impact of different anticoagulant strategies and to identify modifiable determinants of cognitive decline in atrial fibrillation; such studies should include a validated frailty assessment alongside cognitive and functional evaluation.

## 5. Conclusions

In conclusion, in this exploratory study of hospitalized patients with atrial fibrillation, cognitive impairment was common; age was consistently associated with lower cognitive and functional scores, and previous ischemic stroke with lower MMSE scores. No association was detected for CRP or LDL-C with cognitive scores, or for diabetes mellitus, sex or oral anticoagulant therapy, although the study was not powered to exclude such associations. Cognitive impairment was closely associated with reduced functional independence. These hypothesis-generating findings warrant confirmation in larger prospective studies, which should also determine whether routine cognitive screening and optimized preventive strategies can reduce the burden of cognitive decline in atrial fibrillation.

## Figures and Tables

**Table 1:** Baseline demographic, clinical, and laboratory characteristics of the study population, overall and by sex.

Characteristic	Overall (*n* = 62)	Women (*n* = 34)	Men (*n* = 28)	*p*-Value
Age, years	75.32 ± 8.08	77.82 ± 6.98	72.29 ± 8.52	**<0.01**
**Atrial fibrillation subtype, *n* (%)**				0.23 ^b^
First diagnosed	4 (6.5)	2 (5.9)	2 (7.1)	
Paroxysmal	16 (25.8)	10 (29.4)	6 (21.4)	
Persistent	6 (9.7)	1 (2.9)	5 (17.9)	
Permanent	34 (54.8)	19 (55.9)	15 (53.6)	
Pharmacologically converted	2 (3.2)	2 (5.9)	0 (0.0)	
**Comorbidities, *n* (%)**				
Previous ischemic stroke	17 (27.4)	14 (41.2)	3 (10.7)	**<0.01** ^b^
Diabetes mellitus	18 (29.0)	8 (23.5)	10 (35.7)	0.29 ^b^
**Education, *n* (%)**				0.28 ^b^
Middle school	7 (11.3)	4 (11.8)	3 (10.7)	
Professional school	36 (58.1)	23 (67.6)	13 (46.4)	
High school	17 (27.4)	6 (17.6)	11 (39.3)	
University	2 (3.2)	1 (2.9)	1 (3.6)	
**Residence, *n* (%)**				0.46 ^b^
Rural	39 (62.9)	20 (58.8)	19 (67.9)	
Urban	23 (37.1)	14 (41.2)	9 (32.1)	
**LVEF, *n* (%)**				**<0.01** ^b^
<40%	11 (17.7)	3 (8.8)	8 (28.6)	
40–49%	25 (40.3)	11 (32.4)	14 (50.0)	
≥50%	26 (41.9)	20 (58.8)	6 (21.4)	
**Cognitive and functional scores**				
MoCA score	17.29 ± 6.16	16.74 ± 6.71	17.96 ± 5.47	0.46 ^a^
MMSE score	21.27 ± 5.94	20.56 ± 6.22	22.14 ± 5.56	0.38 ^a^
ADL score	7.87 ± 2.61	7.68 ± 2.74	8.11 ± 2.48	0.66 ^a^
**Laboratory parameters**				
LDL-C, mg/dL	80.66 ± 34.73	77.40 ± 27.70	84.62 ± 41.89	0.64 ^a^
CRP, mg/L—median (IQR)	6.5 (2.9–28.6)	4.7 (1.8–11.6)	12.6 (4.8–62.1)	**0.02** ^a^
**Anticoagulant class, *n* (%)**				0.81 ^b^
Vitamin K antagonist (acenocoumarol)	5 (8.1)	3 (8.8)	2 (7.1)	
Direct oral anticoagulant	57 (91.9)	31 (91.2)	26 (92.9)	
**Anticoagulant agent, *n* (%)**				0.97 ^b^
Acenocoumarol	5 (8.1)	3 (8.8)	2 (7.1)	
Apixaban	41 (66.1)	23 (67.6)	18 (64.3)	
Edoxaban	12 (19.4)	6 (17.6)	6 (21.4)	
Rivaroxaban	4 (6.5)	2 (5.9)	2 (7.1)	

**Abbreviations:** ADL, Activities of Daily Living; CRP, C-reactive protein; LDL-C, low-density lipoprotein cholesterol; MMSE, Mini-Mental State Examination; MoCA, Montreal Cognitive Assessment; SD, standard deviation. Normally distributed continuous variables are presented as mean ± SD and non-normally distributed variables as median (interquartile range); categorical variables are presented as *n* (%). Percentages are column percentages and may not total 100 because of rounding. *p*-Values refer to the comparison between women and men. ^a^ Mann–Whitney U test. ^b^ Chi-square test. Statistical significance was set at *p* < 0.05; significant values are shown in bold.

**Table 2:** Cognitive and functional performance according to history of previous ischemic stroke.

Outcome	No Previous Stroke (*n* = 45)	Previous Stroke (*n* = 17)	Mean Difference (95% CI)	*p*-Value (q)	Cohen’s *d* (95% CI)
MoCA score	18.42 ± 5.58	14.29 ± 6.80	4.13 (0.75 to 7.51)	0.02 (0.022)	0.70 (0.13 to 1.27)
MMSE score	22.60 ± 5.20	17.76 ± 6.48	4.84 (1.67 to 8.01)	<0.01 (0.010)	0.87 (0.29 to 1.45)
ADL score	8.33 ± 2.33	6.65 ± 3.00	1.69 (0.25 to 3.12)	0.02 (0.022)	0.67 (0.10 to 1.24)

**Abbreviations:** ADL, Activities of Daily Living; CI, confidence interval; MMSE, Mini-Mental State Examination; MoCA, Montreal Cognitive Assessment; SD, standard deviation. Values in the group columns are mean ± SD. The mean difference is calculated as the no-previous-stroke group minus the previous-stroke group; positive values indicate better performance in patients without previous stroke. Groups were compared using the independent-samples *t*-test. Cohen’s d was calculated using the pooled standard deviation; values of 0.2, 0.5 and 0.8 conventionally denote small, moderate and large effects. Statistical significance was set at *p* < 0.05. q-Values are Benjamini–Hochberg false-discovery-rate-adjusted *p*-values across the three comparisons in this table.

**Table 3:** Spearman’s rank correlations between biomarkers, cognitive performance, and functional status.

Variable	MoCA Score		MMSE Score		ADL Score	
	ρ (95% CI)	*p* (q)	ρ (95% CI)	*p* (q)	ρ (95% CI)	*p* (q)
CHA_2_DS_2_-VASc	−0.35 (−0.56 to −0.09)	0.006 (0.017)	−0.36 (−0.57 to −0.11)	0.004 (0.014)	−0.36 (−0.57 to −0.11)	0.004 (0.014)
Education (years)	0.24 (−0.02 to 0.48)	0.057 (0.102)	0.30 (0.04 to 0.52)	0.019 (0.042)	0.11 (−0.16 to 0.37)	0.38 (0.430)
LVEF (%)	0.12 (−0.14 to 0.38)	0.34 (0.430)	0.13 (−0.14 to 0.38)	0.31 (0.430)	0.26 (−0.00 to 0.49)	0.039 (0.077)
CRP (mg/L)	−0.12 (−0.37 to 0.15)	0.36 (0.430)	−0.13 (−0.38 to 0.14)	0.32 (0.430)	−0.31 (−0.53 to −0.05)	0.015 (0.039)
LDL-C (mg/dL)	0.11 (−0.16 to 0.37)	0.38 (0.430)	0.01 (−0.25 to 0.28)	0.92 (0.920)	0.09 (−0.18 to 0.34)	0.51 (0.535)
MoCA score	—	—	0.86 (0.77 to 0.92)	<0.001 (<0.001)	0.49 (0.26 to 0.67)	<0.001 (<0.001)
MMSE score	0.86 (0.77 to 0.92)	<0.001 (<0.001)	—	—	0.50 (0.27 to 0.67)	<0.001 (<0.001)

**Abbreviations:** ADL, Activities of Daily Living; CRP, C-reactive protein; LDL-C, low-density lipoprotein cholesterol; MMSE, Mini-Mental State Examination; MoCA, Montreal Cognitive Assessment. ρ denotes the Spearman’s rank correlation coefficient, with 95% confidence intervals derived by Fisher z transformation. Em dashes indicate the correlation of a variable with itself. Statistical significance was set at *p* < 0.05. q-Values (in parentheses) are Benjamini–Hochberg false-discovery-rate-adjusted *p*-values across all correlations in this table.

**Table 4:** Multivariable linear regression analysis of predictors of cognitive and functional performance.

Predictor	MoCA Score		MMSE Score		ADL Score	
	β (95% CI)	*p* (q)	β (95% CI)	*p* (q)	β (95% CI)	*p* (q)
Age, per year	−0.37 (−0.56 to −0.17)	<0.001 (0.006)	−0.34 (−0.51 to −0.16)	<0.001 (0.006)	−0.14 (−0.23 to −0.05)	0.003 (0.023)
Previous ischemic stroke	−3.94 (−7.42 to −0.46)	0.027 (0.130)	−4.59 (−7.74 to −1.44)	0.005 (0.030)	−1.36 (−2.93 to 0.20)	0.087 (0.348)
CRP, per 10 mg/L	−0.20 (−0.51 to 0.12)	0.21 (0.629)	−0.19 (−0.47 to 0.10)	0.19 (0.629)	−0.05 (−0.20 to 0.09)	0.45 (0.651)
LDL-C, per 10 mg/dL	−0.04 (−0.48 to 0.39)	0.85 (0.923)	−0.14 (−0.54 to 0.25)	0.47 (0.651)	−0.01 (−0.20 to 0.19)	0.95 (0.981)
Education, per year	0.12 (−0.86 to 1.10)	0.81 (0.923)	0.33 (−0.56 to 1.22)	0.46 (0.651)	−0.01 (−0.45 to 0.43)	0.98 (0.981)
Male sex (reference: female)	−1.53 (−4.79 to 1.72)	0.35 (0.651)	−1.31 (−4.26 to 1.63)	0.38 (0.651)	−0.51 (−1.97 to 0.96)	0.49 (0.651)
Diabetes mellitus	0.57 (−2.84 to 3.99)	0.74 (0.885)	1.24 (−1.85 to 4.32)	0.43 (0.651)	−0.54 (−2.08 to 0.99)	0.48 (0.651)
DOAC vs. vitamin K antagonist	−1.10 (−6.52 to 4.33)	0.69 (0.868)	−2.68 (−7.59 to 2.23)	0.28 (0.651)	−1.13 (−3.57 to 1.31)	0.36 (0.651)

**Abbreviations:** ADL, Activities of Daily Living; CRP, C-reactive protein; DOAC, direct oral anticoagulant; LDL-C, low-density lipoprotein cholesterol; MMSE, Mini-Mental State Examination; MoCA, Montreal Cognitive Assessment. β denotes the unstandardized regression coefficient with its 95% confidence interval. Coefficients for CRP and LDL-C are expressed per 10-unit increment. Each column represents a separate model adjusted for all eight listed predictors. Model performance is reported in Table 5. Statistical significance was set at *p* < 0.05. q-Values (in parentheses) are Benjamini–Hochberg false-discovery-rate-adjusted *p*-values across all 24 coefficients in this table.

**Table 5:** Performance of the multivariable linear regression models.

Outcome	R^2^ (Optimism-Corrected)	Adjusted R^2^	RMSE
MoCA score	0.35 (0.16)	0.25	5.34
MMSE score	0.42 (0.25)	0.34	4.84
ADL score	0.27 (0.06)	0.15	2.40

**Abbreviations:** ADL, Activities of Daily Living; MMSE, Mini-Mental State Examination; MoCA, Montreal Cognitive Assessment; RMSE, root mean square error. R^2^ denotes the coefficient of determination. Models correspond to those presented in Table 4. Optimism-corrected R^2^ (in parentheses) was estimated by bootstrap resampling (2000 resamples).

**Table 6:** CHA_2_DS_2_-VASc score as a predictor of cognitive and functional performance.

Outcome	β per Point	95% CI	*p* (q)	R^2^	RMSE
**Unadjusted**					
MoCA score	−1.30	−2.16 to −0.44	0.004 (0.004)	0.13	5.79
MMSE score	−1.29	−2.11 to −0.46	0.003 (0.003)	0.14	5.55
ADL score	−0.57	−0.93 to −0.20	0.003 (0.003)	0.14	2.45
**Adjusted for CRP and LDL-C**					
MoCA score	−1.39	−2.28 to −0.50	0.003 (0.003)	0.17	5.77
MMSE score	−1.44	−2.28 to −0.59	0.001 (0.003)	0.18	5.50
ADL score	−0.59	−0.97 to −0.21	0.003 (0.003)	0.16	2.46

**Abbreviations:** ADL, Activities of Daily Living; CI, confidence interval; CRP, C-reactive protein; CHA2DS2-VASc, congestive heart failure, hypertension, age, diabetes, stroke, vascular disease, sex category; LDL-C, low-density lipoprotein cholesterol; MMSE, Mini-Mental State Examination; MoCA, Montreal Cognitive Assessment; RMSE, root mean square error. β denotes the unstandardized regression coefficient per one-point increase in the score. Age, diabetes mellitus, previous stroke and female sex are components of the score and were therefore not included as covariates; CRP and LDL-C are the only available non-component variables. Statistical significance was set at *p* < 0.05. q-Values (in parentheses) are Benjamini–Hochberg false-discovery-rate-adjusted *p*-values across the six models in this table.

## Data Availability

The data supporting the findings of this study are available from the corresponding author upon reasonable request. The data are not publicly available because they contain information that could compromise the privacy of research participants.

## Supplementary Materials

The following supporting information can be downloaded at https://www.mdpi.com/article/10.3390/jcm15208075/s1, Table S1: Parsimonious multivariable models; Table S2: Penalized regression on the eight predictors of the primary model; Table S3: Bootstrap stability and optimism-corrected model performance; Table S4: Models including AF subtype and left ventricular ejection fraction; Table S5: Sensitivity analyses for C-reactive protein; Table S6: Regression diagnostics and robust estimates; Table S7: Place of residence adjusted for age, education and previous stroke.

## Abbreviations

ADL, Activities of Daily Living; AF, atrial fibrillation; CI, confidence interval; CRP, C-reactive protein; DOAC, direct oral anticoagulant; FDR, false discovery rate; IQR, interquartile range; LASSO, least absolute shrinkage and selection operator; LDL-C, low-density lipoprotein cholesterol; LVEF, left ventricular ejection fraction; MMSE, Mini-Mental State Examination; MoCA, Montreal Cognitive Assessment; RMSE, root mean square error; SD, standard deviation.
