# Supplementary material for: Cognitive Impairment and Functional Independence in Hospitalized Patients with Atrial Fibrillation: A Cross-Sectional Study
Source: J Clin Med. 2026 Oct 17;15(20):8075. doi: 10.3390/jcm15208075 (PMC13706352; doi:10.3390/jcm15208075)
Supplement: Supplementary file 1 [file jcm-15-08075-s001.zip › jcm-4558945-supplementary.pdf]

Supplementary Materials

Cognitive Impairment and Functional Independence in Hospitalized Patients with Atrial Fibrillation: A Cross-Sectional Study

Table S1. Parsimonious multivariable models (four predictors; 15.5 participants per predictor).

Predictor	MoCA β (95% CI)	p	MMSE β (95% CI)	p	ADL β (95% CI)	p
Age, per year	−0.36 (−0.55 to −0.17)	<0.001	−0.33 (−0.50 to −0.15)	<0.001	−0.13 (−0.22 to −0.05)	0.003
Previous ischemic stroke	−3.88 (−7.07 to −0.69)	0.018	−4.54 (−7.52 to −1.56)	0.003	−1.63 (−3.06 to −0.20)	0.027
Education, per year	0.27 (−0.66 to 1.20)	0.567	0.45 (−0.42 to 1.32)	0.301	0.04 (−0.38 to 0.46)	0.858
Male sex (reference: female)	−2.09 (−5.10 to 0.91)	0.169	−1.83 (−4.64 to 0.98)	0.198	−0.81 (−2.16 to 0.54)	0.236
Adjusted R^2	0.27		0.32		0.18	

Linear regression with MoCA, MMSE and ADL scores as dependent variables, each model including age, previous ischemic stroke, educational attainment and sex. β, unstandardized regression coefficient; CI, confidence interval.

Table S2. Penalized regression on the eight predictors of the primary model.

Predictor	LASSO coefficient	Selected by LASSO in bootstrap resamples (%)	Ridge coefficient
MoCA score			
Age, per year	−2.41	99	−1.98
Previous ischemic stroke	−1.09	90	−1.19
CRP, per 10 mg/L	−0.65	78	−0.66
LDL-C, per 10 mg/dL	0 (excluded)	49	0.02
Education, per year	0 (excluded)	47	0.41
Male sex (reference: female)	0 (excluded)	53	−0.26
Diabetes mellitus	0 (excluded)	47	0.18
DOAC vs. vitamin K antagonist	−0.06	56	−0.31
MMSE score			
Age, per year	−2.20	100	−1.94
Previous ischemic stroke	−1.34	97	−1.47
CRP, per 10 mg/L	−0.52	81	−0.67
LDL-C, per 10 mg/dL	0 (excluded)	61	−0.28
Education, per year	0.24	74	0.61
Male sex (reference: female)	0 (excluded)	59	−0.22
Diabetes mellitus	0.14	69	0.43
DOAC vs. vitamin K antagonist	−0.60	87	−0.67
ADL score			
Age, per year	−0.66	97	−0.58
Previous ischemic stroke	−0.31	80	−0.37
CRP, per 10 mg/L	0 (excluded)	57	−0.16
LDL-C, per 10 mg/dL	0 (excluded)	51	0.06
Education, per year	0 (excluded)	42	0.11
Male sex (reference: female)	0 (excluded)	50	−0.04
Diabetes mellitus	0 (excluded)	50	−0.12
DOAC vs. vitamin K antagonist	0 (excluded)	66	−0.19

Coefficients are standardized (per 1 SD of each predictor). The LASSO penalty was selected by repeated five-fold cross-validation (10 repeats); selection frequency was estimated across 300 bootstrap resamples, each with its own cross-validated penalty. Ridge penalty selected by cross-validation over 60 values.

Table S3. Bootstrap stability of coefficients and optimism-corrected model performance.

A. Stability of the coefficients for age and previous stroke (2000 bootstrap resamples)

Outcome	Predictor	Coefficient negative (%)	p < 0.05 (%)	Bootstrap percentile 95% CI
MoCA score	Age, per year	100	97	−0.60 to −0.20
	Previous ischemic stroke	97	58	−7.56 to 0.12
MMSE score	Age, per year	100	98	−0.54 to −0.19
	Previous ischemic stroke	100	79	−7.77 to −1.01
ADL score	Age, per year	100	91	−0.24 to −0.07
	Previous ischemic stroke	92	38	−2.96 to 0.50

B. Model performance

Outcome	Apparent R^2	Adjusted R^2	Optimism-corrected R^2
MoCA score	0.35	0.25	0.16
MMSE score	0.42	0.34	0.25
ADL score	0.27	0.15	0.06

Optimism was estimated as the mean difference between each bootstrap model's R^2 in its bootstrap sample and in the original data, and subtracted from the apparent R^2.

Table S4. Primary models with additional adjustment for AF subtype or left ventricular ejection fraction.

A. Adding permanent versus non-permanent AF

Predictor	MoCA β (95% CI)	p	MMSE β (95% CI)	p	ADL β (95% CI)	p
Age, per year	−0.36 (−0.56 to −0.15)	<0.001	−0.33 (−0.51 to −0.14)	<0.001	−0.15 (−0.24 to −0.06)	0.001
Previous ischemic stroke	−3.87 (−7.38 to −0.35)	0.032	−4.53 (−7.71 to −1.35)	0.006	−1.45 (−3.00 to 0.10)	0.067
Permanent AF	0.86 (−2.18 to 3.89)	0.573	0.66 (−2.09 to 3.41)	0.633	−1.00 (−2.34 to 0.34)	0.141

B. Adding LVEF below 40%

Predictor	MoCA β (95% CI)	p	MMSE β (95% CI)	p	ADL β (95% CI)	p
Age, per year	−0.35 (−0.55 to −0.16)	<0.001	−0.32 (−0.50 to −0.15)	<0.001	−0.13 (−0.22 to −0.05)	0.003
Previous ischemic stroke	−4.44 (−7.89 to −0.98)	0.013	−5.00 (−8.14 to −1.85)	0.002	−1.61 (−3.16 to −0.07)	0.041
LVEF < 40%	−3.60 (−7.63 to 0.44)	0.080	−2.92 (−6.60 to 0.75)	0.117	−1.83 (−3.63 to −0.02)	0.047

Each model includes the eight predictors of the primary model plus the variable shown; only age, previous stroke and the added variable are displayed. LVEF, left ventricular ejection fraction.

Table S5. Sensitivity analyses for C-reactive protein.

A. Primary model with log-transformed CRP (n = 62)

Predictor	MoCA β (95% CI)	p	MMSE β (95% CI)	p	ADL β (95% CI)	p
Age, per year	−0.35 (−0.55 to −0.16)	<0.001	−0.32 (−0.50 to −0.14)	<0.001	−0.13 (−0.22 to −0.04)	0.004
Previous ischemic stroke	−3.77 (−7.24 to −0.29)	0.034	−4.43 (−7.51 to −1.34)	0.006	−1.32 (−2.81 to 0.18)	0.084
CRP, per log unit	−0.54 (−1.42 to 0.35)	0.229	−0.73 (−1.52 to 0.05)	0.067	−0.43 (−0.82 to −0.05)	0.027
LDL-C, per 10 mg/dL	−0.05 (−0.49 to 0.39)	0.821	−0.17 (−0.56 to 0.22)	0.379	−0.04 (−0.23 to 0.15)	0.691

B. Participants with CRP ≤ 10 mg/L (n = 37)

Outcome	Spearman ρ	p	Multivariable β for CRP, per mg/L (95% CI)	p
MoCA score	−0.11	0.526	−0.20 (−1.02 to 0.62)	0.626
MMSE score	−0.14	0.411	−0.28 (−0.97 to 0.41)	0.416
ADL score	−0.43	0.008	−0.56 (−0.85 to −0.26)	<0.001

Panel B excludes participants with CRP above 10 mg/L, whose values may reflect an acute-phase response; the multivariable model includes the eight primary predictors, with CRP entered per mg/L. These analyses were not prespecified, and the subset model has approximately 4.6 participants per predictor; they are exploratory.

Table S6. Regression diagnostics and robust estimates for the primary models.

Diagnostic	MoCA	MMSE	ADL
Shapiro–Wilk test of residuals, p	0.145	0.421	<0.001
Breusch–Pagan test, p	0.836	0.914	0.423
RESET test, p	0.074	0.016	0.193
RESET test with quadratic age term, p	0.655	0.150	0.335
Quadratic age term, p	0.118	0.113	0.564
Maximum Cook's distance	0.11	0.23	0.20
Age, p with HC3 robust SE	<0.001	<0.001	0.003
Previous stroke, p with HC3 robust SE	0.056	0.016	0.165

Maximum variance inflation factor across predictors: 1.42. HC3, heteroscedasticity-consistent standard errors. Non-normal ADL residuals reflect a ceiling effect (22 of 62 participants scored the maximum of 10); bootstrap percentile intervals for ADL are given in Table S3.

Table S7. Place of residence and cognitive and functional performance.

Outcome	Crude β for rural residence (95% CI)	p	Adjusted β for rural residence (95% CI)	p
MoCA score	−2.93 (−6.11 to 0.26)	0.071	−4.15 (−7.13 to −1.18)	0.007
MMSE score	−1.22 (−4.36 to 1.91)	0.438	−1.99 (−4.90 to 0.92)	0.175
ADL score	−0.55 (−1.93 to 0.83)	0.428	−1.01 (−2.41 to 0.38)	0.151

Reference category: urban residence. Adjusted models include age, educational attainment (years) and previous ischemic stroke. This analysis was not prespecified and is exploratory.
